# Urban geography and scaling of contemporary Indian cities

**DOI:** 10.1098/rsif.2018.0758

**Published:** 2019-03-13

**Authors:** Anand Sahasranaman, Luís M. A. Bettencourt

**Affiliations:** 1 Centre for Complexity Science, Department of Mathematics, Imperial College London, London SW72AZ, UK; 2 Division of Mathematics and Computer Science, Krea University, Sri City, Andhra Pradesh 517646, India; 3 Mansueto Institute for Urban Innovation, Department of Ecology and Evolution, Department of Sociology, University of Chicago, Chicago, IL 60637, USA; 4 Santa Fe Institute, Santa Fe, NM 87501, USA

**Keywords:** urban development, density, innovation, crime, infrastructure, urban agglomeration

## Abstract

This paper attempts to create a first comprehensive analysis of the integrated characteristics of contemporary Indian cities, using scaling and geographical analysis over a set of diverse indicators. We use data of *urban agglomerations* in India from the Census 2011 and from a few other sources to characterize patterns of urban population density, infrastructure, urban services, crime and technological innovation. Many of the results are in line with expectations from urban theory and with the behaviour of analogous quantities in other urban systems in both high and middle-income nations. India is a continental scale, fast developing urban system, and consequently there are also a number of interesting exceptions and surprises related to both particular quantities and strong regional patterns of variation. Specifically, these relate to the potential salience of gender and caste in driving sub-linear scaling of crime and to the geography of technological innovation. We characterize these patterns in detail for crime and invention, and connect them to the existing literature on their determinants in a specifically Indian context. The paucity of data at the urban level and the absence of official definitions for functional cities in India create a number of limitations and caveats to any present analysis. We discuss these shortcomings and spell out the challenge for a systematic statistical data collection relevant to cities and urban development in India.

## 1. Introduction

In 2007, for the first time in history, the world population became more urban than rural. This phenomenon is only expected to intensify, with the UN projecting that 66% of the global population will live in cities by 2050, and that over 90% of this increase is expected to be focused in Asia and Africa [1]. Given this context of rapidly increasing urbanization, especially in the developing world, there is an urgency to develop a deeper, scientific understanding of urban processes and their practical implications.

Against this general backdrop of worldwide urbanization, one particular country—India—accounts for the most momentous transformation of all. India is on track to becoming the largest nation in the world by population, expected to surpass China in the next few years with a population around 1.4 billion by 2024 [2]. India's population is expected to peak in the range 1.6–1.8 billion by mid-century. All along, the nation will continue to urbanize from a rate close to 33% today to more than 50% in the same time frame [2]. This translates into approximately an additional 400 million people living in Indian cities in the next three decades, placing pressure on land, urban services, governance structures and general economic development, all of which will need the be substantially transformed very quickly.

The prospect of guiding this massive urbanization successfully in the next few decades demands a much deeper assessment and understanding of Indian cities and their trajectories. Although there is a rich history of case studies, demographic analyses [3–5] and some detailed investigations of specific quantities such as sanitation, slums, and crime [6–10], the present paper—to the best of our knowledge—is the first detailed analysis of Indian cities as complex systems, where a quantitative assessment of many urban attributes is brought together into a common framework.

To do this, we use the framework of urban scaling, together with ideas from urban geography [11–14]. Urban scaling analysis singles out the importance of population size in isolating a set of general agglomeration effects, characterizing economies of scale in infrastructure and urban services, and increasing returns to scale in socioeconomic interactions [11,14]. These effects are the tell-tale signals of cities, and have been observed quantitatively in urban systems from around the world, from the United States to European nations, and from China to South Africa and Brazil [11,12,15–18].

The starting point of the analysis is very simple: any extensive urban indicator *Y_i_*(*t*, *N_i_*) for city *i*, with population size *N_i_*(*t*), at time *t* can be described as:

1.1 $$Y_{i}(t,\;N_{i})=Y_{0}(t)N_{i}{\left(t\right)}^{\beta }e^{\xi_{i}(t)},\;\;\;\;\;\;$$

which is an exact expression. The prefactor *Y*_0_(*t*) is independent of size and carries the physical dimensions of the relevant quantity (e.g. money per year for gross domestic product (GDP)). Its change in time signifies systemic change for all cities, such as national level economic growth. The scaling exponent *β* is the elasticity of the quantity *Y_i_* relative to population at fixed *t*,

1.2 $$\beta =\frac{d\ln Y_{i}}{d\ln N_{i}}.$$

The quantities *ξ_i_*(*t*) are city specific, scale independent deviations from the scaling law, also known as scale-adjusted metropolitan indicators (SAMIs),

1.3 $$\xi_{i}(t)=\ln\frac{Y_{i}(t)}{Y_{0}(t)N_{i}{\left(t\right)}^{\beta }}.$$

The point about scaling analysis is twofold. First, the exponent *β* is observed empirically to take similar values for broad classes of urban indicators [11,14], with *β* ≃ 7/6 > 1, for socioeconomic rates, *β* ≃ 5/6 < 1, for spatial density and several kinds of infrastructure, and *β* ≃ 1, for household quantities expressing typical individual needs (number of jobs, number of housing units, water consumed at home). Second, these specific exponent values can be computed from urban scaling theory [14], which expresses classical models of urban economics and geography in modern terms, including socioeconomic networks and more realistic transportation costs [19–21].

In this way urban scaling theory—like most earlier mathematical models of urban economics and regional science—defines cities through a budget constraint balancing urban incomes and costs of real estate and transportation [14,19]. This definition translates in practice to so-called functional cities, which are urban areas defined as integrated labour markets or ‘commute to work’ areas; that is regions that comprise together places of residence and work, such as business districts and corresponding residential areas and suburbs. In the United States, this definition corresponds to metropolitan statistical areas [22], which have been constructed by the US Census since the 1950s. In OECD countries, these constructions are more recent [23], but have been created at a higher spatial resolution. In India, no similar metropolitan definition of cities exists at the moment, in part because commuting flows remain hard to measure. Instead, at present, the Census of India builds units of analysis known as *urban agglomerations,* which are only an approximation to these concepts. Urban agglomerations are defined by spatial contiguity, under several additional conditions (see electronic supplementary material, appendix A). This is an important caveat because urban units of analysis that are ‘not functional’, and are instead defined through metrics such as density, or by political boundaries, sometimes are found not to display urban agglomeration properties [24,25].

Despite these caveats, which are at present hard to resolve, we believe that the scaling analysis of urban agglomerations is a fundamental first step for a systemic understanding of Indian cities. Below, we explore the scaling relationships of various spatial, socio economic and infrastructural characteristics of Indian cities with their populations. We analyse the geography of exceptions to average scaling and attempt to make sense of these deviations by invoking a deeper understanding of the geography and history of India. Finally, we discuss how data for Indian cities must improve in the near future and point out priorities in light of our results and other general considerations from the emerging field of urban sciences.

## 2. Agglomeration and scaling effects in Indian cities

We start by exploring the nature of scaling relationships for three sets of urban characteristics: public infrastructure, social interactions and individual household needs. We present the detailed discussion on urban units of analysis, data and statistical methods used in electronic supplementary material, appendix A. We structure our results in two parts—the first deals with the analysis of scaling (agglomeration) effects in Indian cities, while the second pays closer attention to two quantities of great interest, namely crime and technological innovation measured via patent counts. Both these quantities show strong regional patterns, which we analyse systematically.

**Figure 1.**
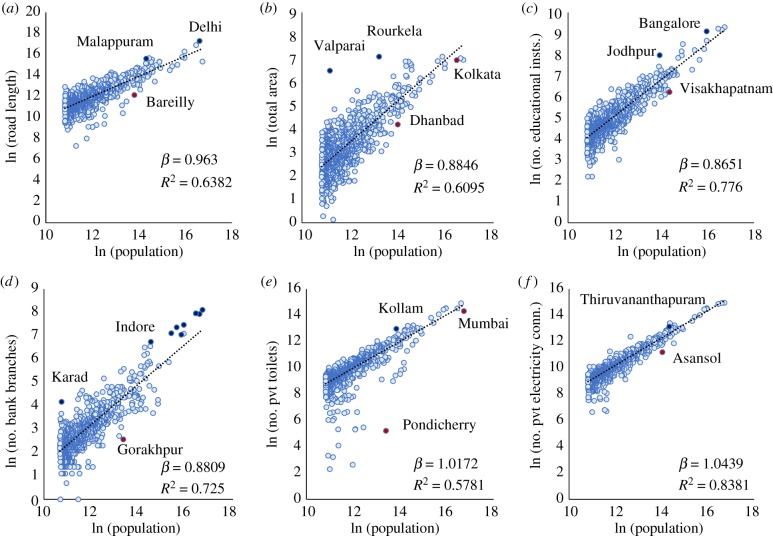
Scaling of public and private infrastructure with population. Each panel shows the total value for each city (light blue circle) and the scaling best fit line, equation (1.1); the best fit estimate for the exponent *β* and the goodness of fit *R*^2^ are also shown, see table 1 and electronic supplementary material, appendix A for details. Urban indicators shown are (*a*) road length, (*b*) total area, (*c*) educational institutions, (*d*) bank branches, (*e*) private toilets and (*f*) private electricity connections. All data from the Census of India 2011, see electronic supplementary material, appendix A for details. (Online version in colour.)

Figure 1 displays a number of scaling diagrams for different public and private types of infrastructure and services.

**Table 1.** The specific statistics of scaling in the Indian context.

*Y*	*β*	95% CI	*R*^2^	number of observations	data
road length	0.96	0.92–1.01	0.64	903	Census 2011
total area	0.88	0.84–0.93	0.61	909	Census 2011
number of educational institutions	0.87	0.83–0.90	0.78	909	Census 2011
number of bank branches	0.88	0.85–0.92	0.72	908	Census 2011
number of private toilets	1.02	0.96–1.07	0.58	909	Census 2011
number of private electricity connections	1.04	1.01–1.07	0.84	909	Census 2011
number of patents	1.53	1.22–1.83	0.95	320	IPI 2004, 2006, 2008, 2011
number of crimes	0.87	0.81–0.93	0.70	317	NCRB 1991, 1996, 2001, 2006, 2011
number of serious crimes (murder and culpable homicide)	0.78	0.69–0.87	0.46	317	NCRB 1991, 1996, 2001, 2006, 2011

First, we find that land area shows a behaviour analogous to other urban systems, historically and throughout the world, and with urban scaling theory (expectation *β* ≃ 5/6), with an observed sublinear exponent *β* ≃ 0.88 (figure 1*b*) [26]. This exponent also sets, according to theory, the length of roads as these should be area filling. However, we find instead a somewhat larger, just barely sublinear, value of *β* ≃ 0.96 (figure 1*a*). It is typical of fast developing nations that formal infrastructure and services are first developed in larger cities and then gradually become more common in smaller places throughout the urban system [15], which may also be the case for India [27]. Other establishments associated with public and social services—such as number (not size) of bank branches and educational institutions—also show sublinear behaviour, roughly tracking land area, suggesting that they occur on average at similar spatial densities in all cities in India big and small, which is again a pattern consistent with other nations. Finally, household services and infrastructure—as measured by private toilets and electricity connections—are roughly proportional (linear) to population, with perhaps just a slightly superlinear effect of a few per cent, possibly accounting for the more universal accessibility of these basic services in larger cities [27]. A few places falling well below these scaling relations point to major local deficits relative to other Indian cities with similar population sizes. Examples are Pondicherry for sanitation, Gorakhpur for banking infrastructure and Visakhapatnam for education infrastructure, to name a few, see figure 1. We also see significant outperformance by the eight largest cities for numbers of bank branches.

As discussed previously, there is some research indicating that scaling indicators can be sensitive to spatial definitions of urban agglomerations [24,25], especially if these are not tied to integrated labour markets and may track instead patterns of political administration or density. To investigate some of these issues in the Indian context given the data presently available, we estimated scaling relationships for public and private infrastructure at the level of individual Indian cities (un-agglomerated). We find scaling exponent estimates that show only minor variations from urban agglomeration values (except private toilets), and that overall, public infrastructure still scales sub-linearly and private infrastructure almost linearly. Further details are available in electronic supplementary material, appendix B.

## 3. Gross domestic product, innovation and crime in Indian cities

First, we consider the classic quantity measuring the size of the economy, the GDP. There are presently no official GDP statistics at the urban agglomeration level from the Government of India or state governments. The lowest level of spatial disaggregation for GDP data appears to be at the district level, which is not specifically urban: large cities span parts of several districts, whereas small cities are contained, together with peri-urban and rural areas, in other districts. Because this investigation is on different units of analysis and requires careful accounting of what is urban, we defer it to future work. For more discussion on this theme, refer to electronic supplementary material, appendix C.

**Figure 2.**
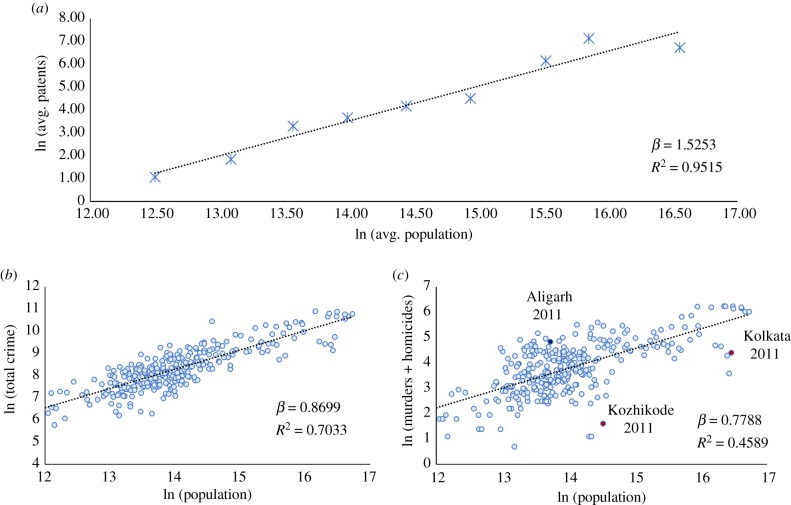
Scaling of technological innovation and crime in Indian urban agglomerations with population. (*a*) Patents filled with the Office of the Controller General of Patents Designs and Trademarks (Intellectual Property India) for the years 2004, 2006, 2008, and 2011, averaged in logarithmic bins. (*b*) Total crime, and (*c*) number of murders and culpable homicides under the Indian Penal Code from the National Crime Records Bureau for the years 1991, 1996, 2001, 2006, and 2011. (Online version in colour.)

We turn to assessing technological innovation rates in Indian cities. The classic quantitative measure for technological innovation is patent applications. However, the annual number of patents is zero for some cities in certain years. To overcome this issue, we use logarithmic binning of cities and data (see electronic supplementary material, appendix A) [28]. Figure 2*a* plots the scaling of patents with population for the population average in each bin. We observe a strongly superlinear relationship with *β* ≃ 1.53. Such a high *β* is in keeping with observations on patent scaling from other jurisdictions (specifically the United States), where the scaling exponent is found to be significantly above 1, though below 1.5 [11,12]. Theoretical work using a network model of the city posits that superlinear scaling for innovation is a robust result (with a wide range of values between 1 and 2 for the exponent), obtainable using fairly loose assumptions [14,29]. For a deeper discussion on the sensitivity of the scaling exponent to techniques used, see electronic supplementary material, appendix D.

Finally, we analyse crime rates in Indian cities. Figure 2*b* shows the scaling of total crimes (defined under the Indian Penal Code) with city population. We find a clear sublinear scaling relationship with population, with an exponent *β* ≃ 0.87. This result stands in stark contrast with the results for crime scaling in other contexts such as the United States [11] and several Latin American nations [16], where there is a superlinear relationship.

It has, however, been argued that total crime data in India is affected by significant under-reporting because of several social and structural factors [30], and that using these data to understand crime in India could yield an unrealistic picture. One subset of crime offences—serious crimes involving loss of life, such as murders and homicides—is posited to be a reasonably accurate measure of reality [16,30]; therefore, we attempt to understand the scaling of such serious crimes with population. Figure 2*c* plots the total numbers of murders and culpable homicides against population. This analysis confirms the sublinear scaling relationship for crime, showing an estimated exponent that is in fact smaller than that for total crime (*β* ≃ 0.78). An observed sublinear scaling versus an expected superlinear trend (proportional to number of interactions) means, of course, that in large Indian cities socialization is substantially more peaceful than in smaller ones (table 1).

In both the case of crime and technological innovation, scaling analysis provides only an approximate picture of urban dynamics. India is a continent-sized nation, with many regional differences, grounded in historic, cultural and political differences across the country. These differences lead to a characteristic urban geography of crime and technological innovation, which we analyse in the next sections.

## 4. The urban geography of crime in India

Deviations from the average population size dependence (scaling) estimated in the previous section, give us a principled way to assess and characterize these local and regional effects [17,31,32]. The residuals from scaling (the vertical distance of each point from the fit line in figures 1 and 2) give us an SAMI, *ξ_i_*(*t*), which characterizes city *i* at time *t:*

4.1 $$\xi_{i}(t)=\ln\frac{Y_{i}(t)}{Y_{0}(t)N_{i}{\left(t\right)}^{\beta }}.$$

This is a dimensionless metric that makes direct comparison between the performance of different cities possible because population size agglomeration effects have been factored out [31].

We now calculate and analyse the SAMIs for patents and crime (only murder and culpable homicide) in India. This will allow us to better assess the nature of local dynamics and also, in the case of crime, potentially explain the counterintuitive observed sublinear scaling effect.

**Figure 3.**
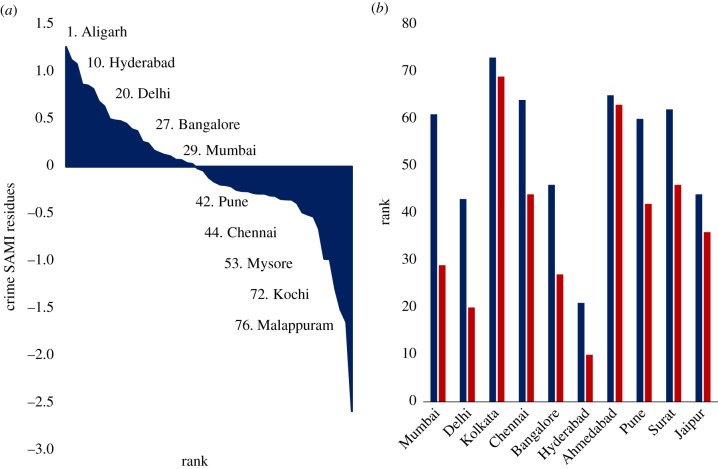
Analysing residuals and per capita metrics for crime. (*a*) Rank order of crime SAMI residues 2011 (*b*) crime rank 2011 by City. Red: crime SAMI residue rank. Blue: crime per capita rank. (Online version in colour.)

Figure 3*a* plots the SAMIs for crime in Indian cities in rank order from more crime than expected to less. Aligarh (Uttar Pradesh) is ranked 1 (worst), while Malappuram (Kerala) is the best performing (safest) city in India. To illustrate the difference between the standard per capita measure of crime (incorporating only murder and culpable homicide) and crime SAMIs (which removes population size bias) we show the expectations from these two approaches for the 10 largest Indian cities. Each one of these cities performs worse when ranked by SAMIs than when measured by a standard crime per capita measure (figure 3*b*). For instance, the per capita crime measure shows us that only one of these 10 cities is in the top half of cities ranked in descending order of crime, while the corresponding SAMI analysis shows that five out of these 10 cities are in the top half. This is because, crime rates being sublinear in population, the SAMIs contain the expectation that larger cities should have less crime per capita and measures only the deviation from such expectation.

Crime in India is arguably an under-researched subject. Studies that explore the theme as a sociological phenomenon do so through the lens of gender [30,33] and caste [34]. The caste system in India today emerges from the ancient ‘varna’ system, where society was divided into five hereditary, endogamous, and occupation-specific groups [34]. The ‘lowest’ castes and the Dalits have historically suffered violence at the hands of the ‘upper’ castes, but post-independence governments in India have attempted to counter this history by expanding the scope of affirmative action and passing specific legislation on crimes against Dalits [34]. While these interventions have resulted in improvements in public goods provision [35] and redistribution of resources [36] to historically disadvantaged groups, social and political organization among Dalits has often resulted in feudal backlashes taking the form of ‘mass killings, gang rapes, looting in Dalit villages’ [37]. Analysis of data from the National Council for Scheduled Castes (NCSC) shows that 60% of the atrocities committed against Dalits in India occurs in four states—Uttar Pradesh, Rajasthan, Bihar and Madhya Pradesh [37]. These data also reveal that most serious crimes against Dalits including hate crimes, rape and murder occur predominantly in rural settings or in small urban settlements.

Gender is the other sociological lens applied to understand crime in India. Dreze & Khera [30] find a robust negative correlation between female to male ratio and murder rates. While causality is hard to assess, they argue that low female–male ratios and high murder rates are both manifestations of patriarchy. This resonates also with the role of evolutionary psychology in the incidence of crime [38]. Given that patriarchal subjugation of women is based on violence (or its implied threat), Dreze & Khera [30] posit that we would indeed expect that areas with high violence would be associated with sharp gender inequalities. This result is also in agreement with Oldenburg [33] on district level data for the northern Indian state of Uttar Pradesh, where he found negative correlation between incidence of murders and female–male ratio. He hypothesized a causal influence of violence on female–male ratios, arguing that in regions of high violence, male child preference would be particularly high as sons are seen both as protection against violence and as potential candidates to exercise power [33]. It is however at the intersection of gender with caste that violent crime becomes salient—in the form of ‘honour crimes’. While ostensibly the legal system in India is free from the grip of caste, there exists a parallel institution of caste-based panchayats (local level clan assemblies) that function based on their own traditional law [39]. Caste panchayats actively rule against inter-caste marriages and even proclaim death as punishment in many cases, also called ‘honour’ killings [39]. While ‘honour’ killings are a pan-Indian phenomenon, it is especially prevalent in the states of northern India—specifically Uttar Pradesh, Delhi, Rajasthan and Haryana [40].

**Figure 4.**
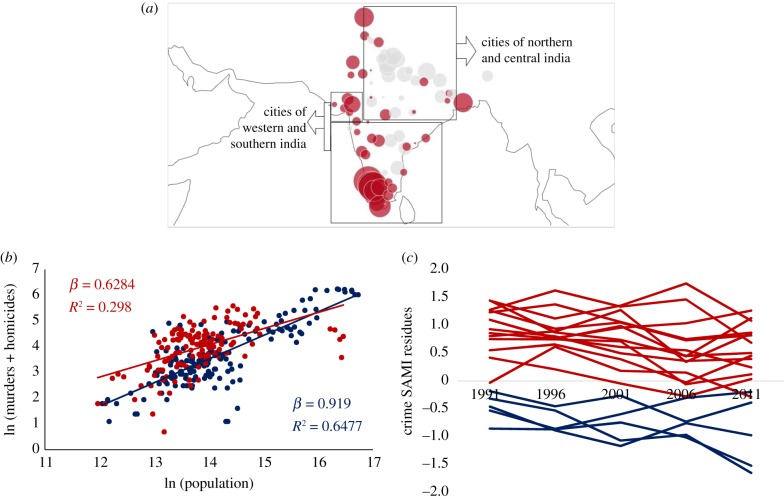
Spatial and temporal analysis of crime residuals. (*a*) Spatial distribution of crime SAMIs in 2011: red (grey) dots correspond to deviations below (above) expectation for city size. The size of the circle denotes the magnitude of crime SAMIs. (*b*) Murders & culpable homicides versus population. Red group: cities in northern and central India. Blue group: cities in southern and western India. (*c*) Temporal evolution of crime SAMIs for select cities between 1991 and 2011. Blue lines: cities in northern and central Indian states of Uttar Pradesh (Agra, Aligarh, Allahabad, Bareilly, Gorakhpur, Kanpur, Lucknow, Meerut, Moradabad, Varanasi), Madhya Pradesh (Bhopal, Indore, Jabalpur), and Bihar (Patna). Red lines: cities in southern Indian states of Kerala (Kochi, Thiruvananthapuram), and Tamil Nadu (Chennai, Coimbatore, Madurai). (Online version in colour.)

We now assess the geographical spread of crime SAMIs to investigate if there is support for these contentions. Figure 4*a* maps the spread of crime SAMIs in India and we can clearly see the distinct geographical tilt in crime behaviour in India. Even a cursory visual inspection makes it clear that a plurality of cities in north and central India, large and small, have high crime SAMIs, while those in western and southern India have much lower crime SAMIs. The higher crime SAMIs in the smaller cities of north-central India, especially given the salience of caste in generating crime, is possibly correlated to the fact that smaller settlements have a higher proportion of Dalit populations. We find evidence for higher Dalit populations in smaller cities when we examine the scaling of Dalit population with city size and discover slightly sublinear scaling with an exponent of 0.96.

We also split the scaling plot for crime into two groups of cities—one group for cities in north and central India and another group for southern and western cities. Figure 4*b* shows that the set of northern-central Indian cities has a significantly lower *β* than the southern-western cities group (0.63 compared to 0.92) because small and medium cities in the northern-central group have higher crime SAMIs than comparable cities in the southern-western set. It is also important to note that even though there is a significant difference in *β* between the two groups, both are still sublinear. This is potentially a reflection of the fact that despite significant differences in crime behaviour across geographies in India, there is indeed an underlying sociology of culture-specific crime (honour and caste-based crime) that is at work across the nation.

These geographical differences also appear to be contingent on history, as evinced in figure 4*c*, which plots the temporal evolution for crime SAMIs for cities in five Indian states between 1991 and 2011. The temporal evolution of crime SAMIs in Uttar Pradesh, Madhya Pradesh and Bihar show that deviations from scaling have remained high throughout this period, while the temporal pattern of crime SAMIs for cities in the southern states of Kerala and Tamil Nadu have remained low. This is an indication that even as cities gain population over decades, relative local characteristics can persist over long time periods [31].

**Figure 5.**
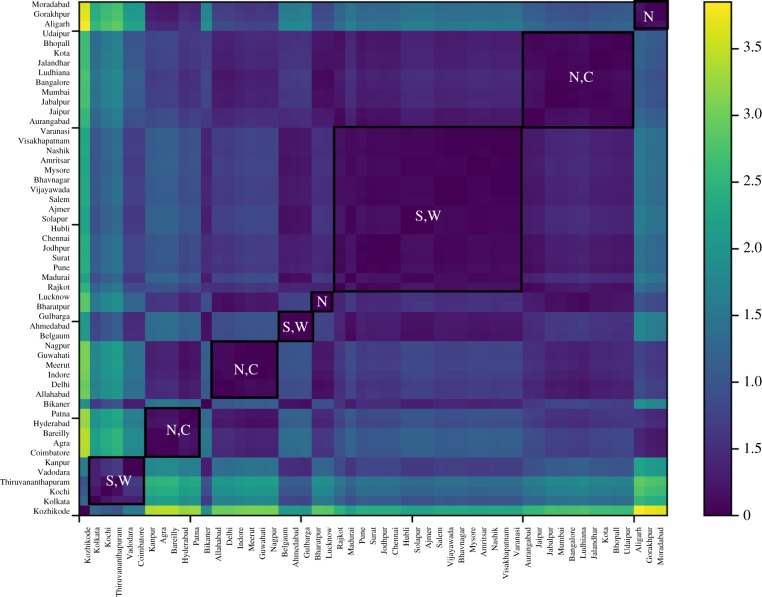
Heatmap of crime SAMIs for Indian cities (2011). Clusters of cities are based on the Euclidean distance between SAMIs (darker blue indicate smaller distances**).** Eight clusters of cities are demarcated and represented by the regions they belong primarily to: N: northern India, C: central India, S: southern India, and W: western India. (Online version in colour.)

We now seek to validate these patterns more formally by attempting to cluster cities based on the distance between their SAMIs. Figure 5 plots a heatmap of the Euclidean distance between pairs of SAMIs. We see eight clusters of cities, with five clusters comprised largely of northern and central Indian cities and the other three clusters of southern and western Indian cities. This decomposition provides a formal confirmation of the spatial spread of SAMIs in figure 4*a* and suggests that regional variations in caste and gender dynamics could be critical to the nature of homicides and murders observed in India.

We also assess how spatial distance affects crime SAMI behaviour. Spatial similarity between cities *i* and *j*, *c_ij_*, is computed as the equal-time cross-correlation of their SAMI time series [31]:

4.2 $$c_{ij}=\frac{1}{\left|\xi_{i}||\xi_{j}\right|}\underset{t}{\sum }\xi_{i}(t)\xi_{j}(t).$$

**Figure 6.**
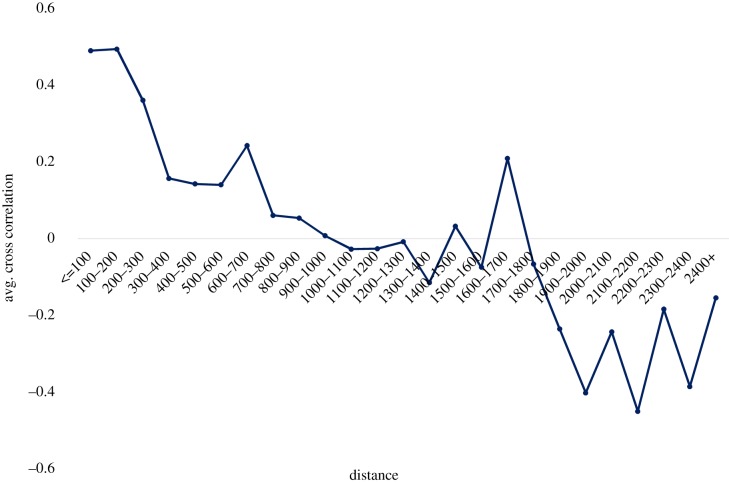
Cross-correlation of crime residues and distance between cities. Average cross-correlation of crime SAMIs of cities (1991–2011) versus straight line distance between cities (in km). (Online version in colour.)

This measure ensures that cities with similar SAMIs and time series have high correlation. Figure 6 plots cross-correlation as a function of distance between cities. We find that while short distance correlation appears to exist for up to approximately 300 km, this effect disappears for greater distances.

To conclude our analysis of crime in Indian cities, we provide some international comparisons to put the Indian evidence into a broader context. UNODC data reveal that, at present, while the global intentional homicide rate is 6.2 (per 100 000 inhabitants), there are significant regional variations, with the Americas and Africa exhibiting high rates of 16.3 and 12.5, and Europe, Oceania, and Asia showing much lower rates of around 3 [41]. Within these regional classifications, there are also significant national variations. The intentional homicide rate in India is found to be 3.2, broadly in line with the Asian average, and lower that of other large countries like Brazil (29.5), Nigeria (9.9) or the United States (5.4), but considerably higher than China (0.6) or Indonesia (0.5). Within nations, when we compare the rates of crime in Indian and American cities, we find that the largest urban agglomerations in India—Mumbai, Delhi and Kolkata—have homicide (murder and culpable homicide) rates of 2.2, 3.0 and 0.6 (see electronic supplementary material, appendix A), while the corresponding homicide rates in the largest metropolitan areas in the United States—New York, Los Angeles and Chicago—are 3.3, 4.9 and 7.1 respectively [42]. Therefore, serious crime in India appears to broadly conform within the regional (Asian) benchmarks, while homicide rates in large Indian cities are significantly lower than their counterparts in the United States today.

## 5. The urban geography of technological innovation in India

**Figure 7.**
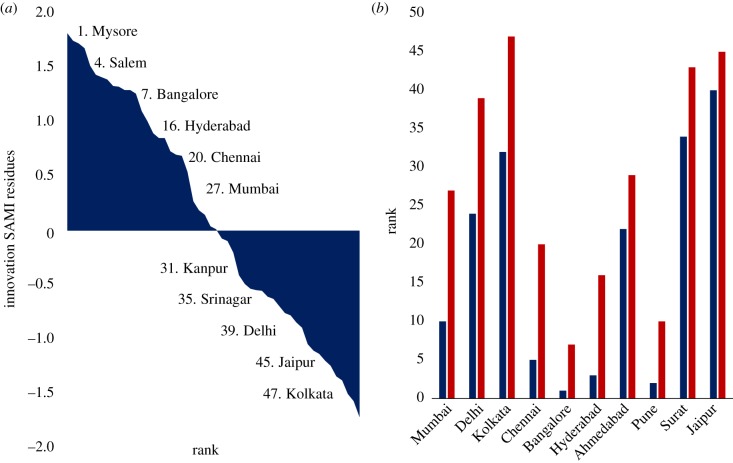
Analyses of scaling residuals and per capita metrics for technological innovation in Indian cities. (*a*) Rank order of innovation SAMI residuals in 2011. (*b*) Technological innovation rank 2011 by city. Red: innovation SAMI residuals rank. Blue: technological innovation per capita rank. (Online version in colour.)

We now turn to an analysis of the deviations in innovation SAMIs, figure 7*a*. Again, we find significant discrepancies between the rank ordering of cities based on innovation SAMIs and technological innovation measured as patents per capita (figure 7*b*). When ordered by patents per capita, five of the 10 largest Indian cities are ranked among the top 10 most inventive cities, but when ranked by innovation SAMI, only one of them, Bangalore, appears in the top 10. This finding points to many medium-sized cities that are quite inventive for their population size and that should be the focus of some additional attention in terms of both scholarship and, where applicable, policy.

**Figure 8.**
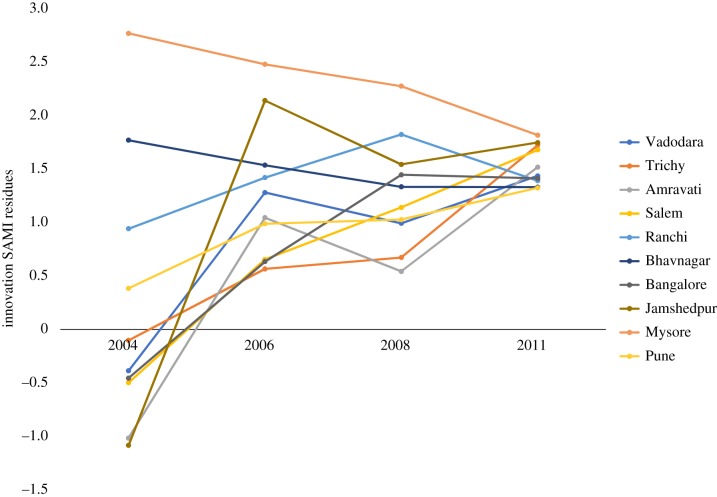
Temporal analysis of innovation scaling residuals (2004–2011) for top 10 cities ranked by SAMIs for patents. (Online version in colour.)

Figure 8 reveals that the 10 most inventive cities in India, once one accounts for strong population size scaling, are in fact predominantly medium-sized cities with a median population of 1.07 million and that their innovation SAMIs have remained consistently high in the period 2006–2011, suggesting temporal persistence of inventive activity. For instance, cities like Jamshedpur, Trichy, Salem and Ranchi, have historically been centres of heavy industry, while Bangalore and Mysore are centres of information technology. Figure 8 also reveals that the SAMI paths of the most inventive cities are converging over time, but such convergence is not in evidence when we consider the temporal SAMI paths of all cities. Therefore, a deeper analysis is required to both understand sectors and drivers of technological innovation across medium-sized outperforming urban centres and also the convergence of their SAMIs over time.

**Figure 9.**
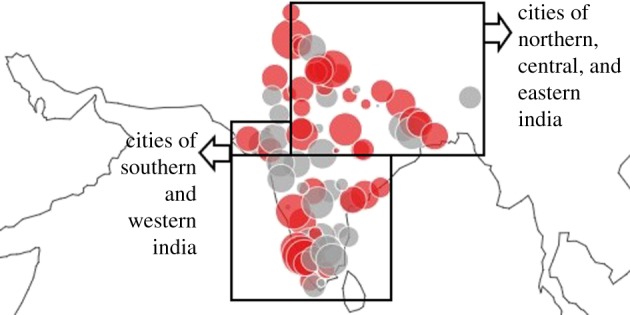
Spatial analysis of innovation SAMI residuals in 2011. Red (grey) dots correspond to deviations below (above) expectation for city size. The size of the circle denotes the magnitude of the corresponding innovation SAMI. (Online version in colour.)

We also map the innovation SAMIs across India in figure 9 and find that spatially, cities in the south and west of India appear generally more likely to be technological innovation hotspots, while cities in the north, centre and east of the country appear to be lagging. However, it is important to point out that while this seems to follow the same overall pattern of crime SAMIs, the extent of regional bias in this case does not appear to be as strong.

**Figure 10.**
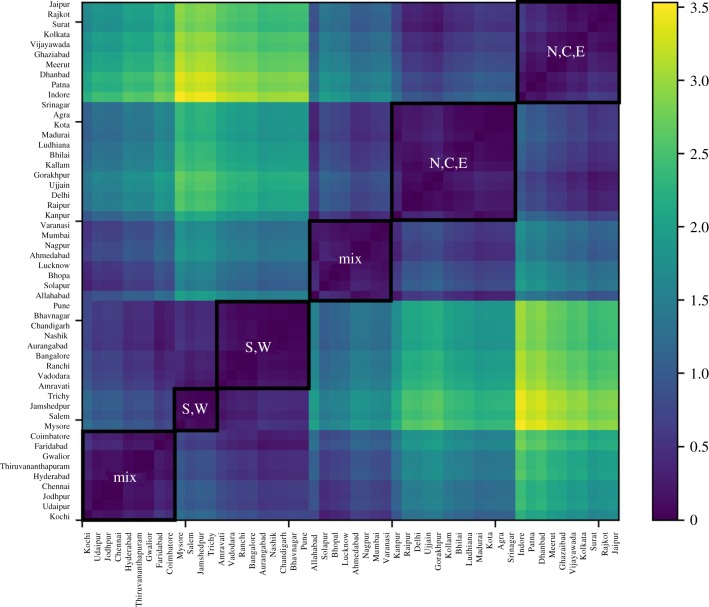
Heatmap of innovation SAMIs of Indian cities (2011). City clusters built based on the Euclidean distance between SAMIs (darker blues indicate lesser distances). Six clusters of cities are demarcated: clusters are represented by N: northern Indian cities, C: central Indian cities, S: southern Indian cities, E: eastern Indian cities and W: western Indian cities. (Online version in colour.)

As before, we tried to confirm this impression by clustering cities based on the Euclidean distance between their innovation SAMI. Figure 10 plots the heatmap of city clusters. It is apparent that, while there are indeed two clusters comprised largely of southern and western cities, and two clusters of northern, central, and eastern cities, there are also two significantly large geographically mixed clusters of cities. So, while there appears to be some extent of regional association, the spatial relationship is not as strong as it was in the case of crime.

## 6. Conclusion

We have analysed emerging data for Indian cities in light of urban theory and known statistical patterns for other urban systems, with the aim of characterizing the tell-tale signals of urbanization in terms of scaling and agglomeration economies, and geographical patterns of development and variation.

Although empirical information at the level of cities is becoming more available in India from official sources, such as the Census (2011), the paucity of data for functional urban definitions relative to most other high- and middle-income countries makes our analysis necessarily limited and tentative. Within these limitations, we find patterns of urban density scaling with population size roughly in line with other urban systems and historical cases. Regarding infrastructure delivery, large Indian cities seem to have an advantage relative to smaller towns, which is a pattern typical of other urban systems where basic infrastructure such as roads, sanitation and electricity access are not yet universal and spread from larger urban areas to other parts of the country [15]. One of the most critical gaps in India is the ability to assess the size and development of urban economies. We discussed existing data and pointed to some contradictions that need to be resolved in order to understand and harness the potential of Indian cities for economic growth. Technological innovation, measured by patenting activity, shows a strong superlinear pattern with city size (similar to other nations, such as the United States), meaning that it is disproportionally concentrated in larger urban areas. Within this general pattern, however, we were able to identify many smaller cities with uncharacteristically high patent productivity and discuss India's detailed geography of technological innovation.

Arguably, the biggest surprise about Indian cities is the sublinear pattern of crime, including murders and homicides, which—unlike in most high-income nations—translates to higher rates of violence per capita in smaller towns, relative to the nation's largest cities. Though many questions about the data remain, we were able to derive the geography of crime across India and relate it to more specific studies that identify most sources of violence in the country associated with issues of gender and caste. These show a strong regional signature and have been discussed by sociologists and anthropologists as a predominantly rural or small city phenomenon.

Indian urbanization, currently estimated at 33%, is expected to rise to 53% by 2050 [2], adding hundreds of millions of people to cities and creating giant megacities, with perhaps as many as 50 million people over that period. While we hope that this paper presents the beginning of a holistic empirical characterization of Indian cities, there is a critical need for a concerted effort aimed at measuring urban economic statistics at the local level, including in neighbourhoods, which tend to express the strongest patterns of concentrated (dis)advantage and thus inequality [15]. Successful Indian urbanization is critical not only for well-being of all people in India, but for the sustainability of the entire planet. We cannot afford to fly blind through this momentous transformation.

## Supplementary Material

Appendices

## Data accessibility

Our paper analyses publicly available datasets from India. The details of the datasets used and their availability are presented in electronic supplementary material: appendix A—Data sources and methods.
