# Supplementary material for: Urban geography and scaling of contemporary Indian cities
Source: J R Soc Interface. 2019 Mar 13;16(152):20180758. doi: 10.1098/rsif.2018.0758 (PMC6451395; doi:10.1098/rsif.2018.0758)
Supplement: Appendices [file rsif20180758supp1.pdf]

Urban Geography and Scaling of Contemporary Indian Cities

Anand Sahasranaman and Luís M. A. Bettencourt

Journal of the Royal Society Interface

APPENDICES

Appendix A: Data sources and methods

Population data: City level population is available from the Census of India at http://www.censusindia.gov.in/2011census/dchb/DCHB.html. To consolidate city level population into population at the Urban Agglomeration (UA) level, the composition of 298 UAs in India is available from the Census of India data, tabulated and provided at http://www.census2011.co.in/urbanagglomeration.php. An *Urban Agglomeration* is defined by the Census of India as "… a continuous urban spread constituting a town and its adjoining urban outgrowths (OGs) or two or more physically contiguous towns together and any adjoining urban outgrowths of such towns.". An urban outgrowth (OG) is defined as ".. a viable unit .. contiguous to a statutory town .. possess(ing) urban features in terms of infrastructure and amenities ..". A complete list of census concepts and definitions is available at http://censusindia.gov.in/2011-prov-results/paper2/data_files/kerala/13-concept-34.pdf. For our analysis, we consider all *urban agglomerations* with populations of at least 50,000 people.

Infrastructure data: Data on city level infrastructure is available from the Census of India at http://www.censusindia.gov.in/2011census/dchb/DCHB.html, under the column "Town Amenities". Each file contains data for a single state. This data is available (for each state) at the city level. aggregation to UA-level data in each state, is just as described for population data. For our scaling analysis, we build the infrastructure metrics from the raw data provided by the Census of India in the following manner:

1. Road length = Pucca (paved) Road Length + Kuccha (unpaved) Road Length
2. Number of educational institutions = Schools (including primary, middle, secondary and senior secondary, both government and private) + Colleges (including arts, science, commerce, arts and science, arts and commerce, arts science and commerce, law, university, medical, engineering, management, and others, both government and private) + Polytechnics (government and private)
3. Number of bank branches = Nationalised bank branches + Private bank branches + Cooperative bank branches
4. Number of private toilets is available as latrine count
5. Number of private electricity collections is also directly provided in raw data
6. Number of commercial and industrial electricity connections = Industrial connections + Commercial connections + Other connections
7. Total Area is directly available in the raw data

For all infrastructure, public and private, we use data from the 2011 census for the scaling analysis. The complete data set has 911 data points.

Gross Domestic Product (GDP) data: There is no official data series of urban GDP in India, so we searched for other sources. We found two small datasets:

1. Price Waterhouse Coopers' 2009 UK Economic Outlook report lists GDP data for 13 Indian cities for 2008. The complete list is available at: https://en.wikipedia.org/wiki/List_of_cities_by_GDP. This data is not drawn from any official series of the Government of India but estimated by PWC. The methodology and approach to estimating GDP is available in Annex B of the report: https://web.archive.org/web/20110504031739/https://www.ukmediacentre.pwc.com/imagelibrary/downloadMedia.ashx?MediaDetailsID=1562. The report estimates GDP at Purchasing Power Parity (PPP) exchange rates to correct for price level differences between countries.
2. McKinsey estimated GDP for 9 Indian cities in 2010. The complete list is available at: https://en.wikipedia.org/wiki/List_of_cities_by_GDP. A sample of this list was published by Foreign Policy titled "The most dynamic cities of 2025", a list of 75 cities around the world. This list contained 2010 GDP estimates for 3 Indian cities and is available at: https://foreignpolicy.com/2012/08/07/the-most-dynamic-cities-of-2025/. This estimate was for nominal GDP and did not incorporate PPP correction.

Crime data: Crime data is released annually at city level by the National Crime Records Bureau (NCRB). These reports, titled "Crime in India", are available online at http://ncrb.gov.in/. Crime is broken down into multiple categories as is common in other nations. For our analysis, we used:

1. Total crime = Total cognizable offences under the Indian Penal Code (IPC)
2. Murders and Homicide = Murder (Sec. 302 IPC) + Culpable Homicide not amounting to murder (Sec. 304 & 308 IPC)

We use crime data for the years 1991, 1996, 2001, 2006, and 2011. While direct census data is used for population numbers in 1991, 2001, and 2011, we interpolate the population numbers for 1996 and 2006 using the Compound Annual Growth Rate (CAGR) calculation for the periods 1991-2001 and 2001-2011 respectively. The complete data set has 317 data points.

Technological Innovation data: We used the published patent records of Intellectual Property India at http://ipindiaservices.gov.in/publicsearch. Given that the data itself is not readily available in a document format, we had to individually search for patent counts on each city. We collect data for the years 2004, 2006, 2008, and 2011. Given the fact that there were a few zero data points (cities with no patents published for a given year), the scaling analysis is not directly performed on all the raw data points. Instead, we bin all the data in logarithmic bins (logbins) of population. For instance, the first logbin of population is 12.25-12.75, which is to say that for all cities whose *log(population)* is between 12.25 and 12.75, their patent counts are averaged, and the logarithm of this average patent count is taken. We therefore end up with 9 logbins of population from 12.25-12.75 to 16.25-16.75 and each of these logbins have a corresponding *log(average patent count)* measure. We plot the scaling relationship between these two derived values to arrive at the scaling exponent for patents (technological innovation, or invention). The complete data set has 320 data points (cities).

Appendix B: Scaling sensitivity to city boundaries

The following analysis, shown in Fig B1, shows scaling plots and estimated exponents for road length, number of educational institutions, number of bank branches, number of private toilets, and number of private electricity connections for un-agglomerated cities. The results of this scaling analysis are as expected from theory, with sub-linear scaling for public infrastructures (road lengths, educational institutions, and bank branches) and linear scaling for private infrastructures (private electricity connections). The only seeming anomaly is the sub-linear scaling of private toilets, which changes to linear scaling upon agglomerating individual cities into approximately functional urban units (Urban Agglomerations).

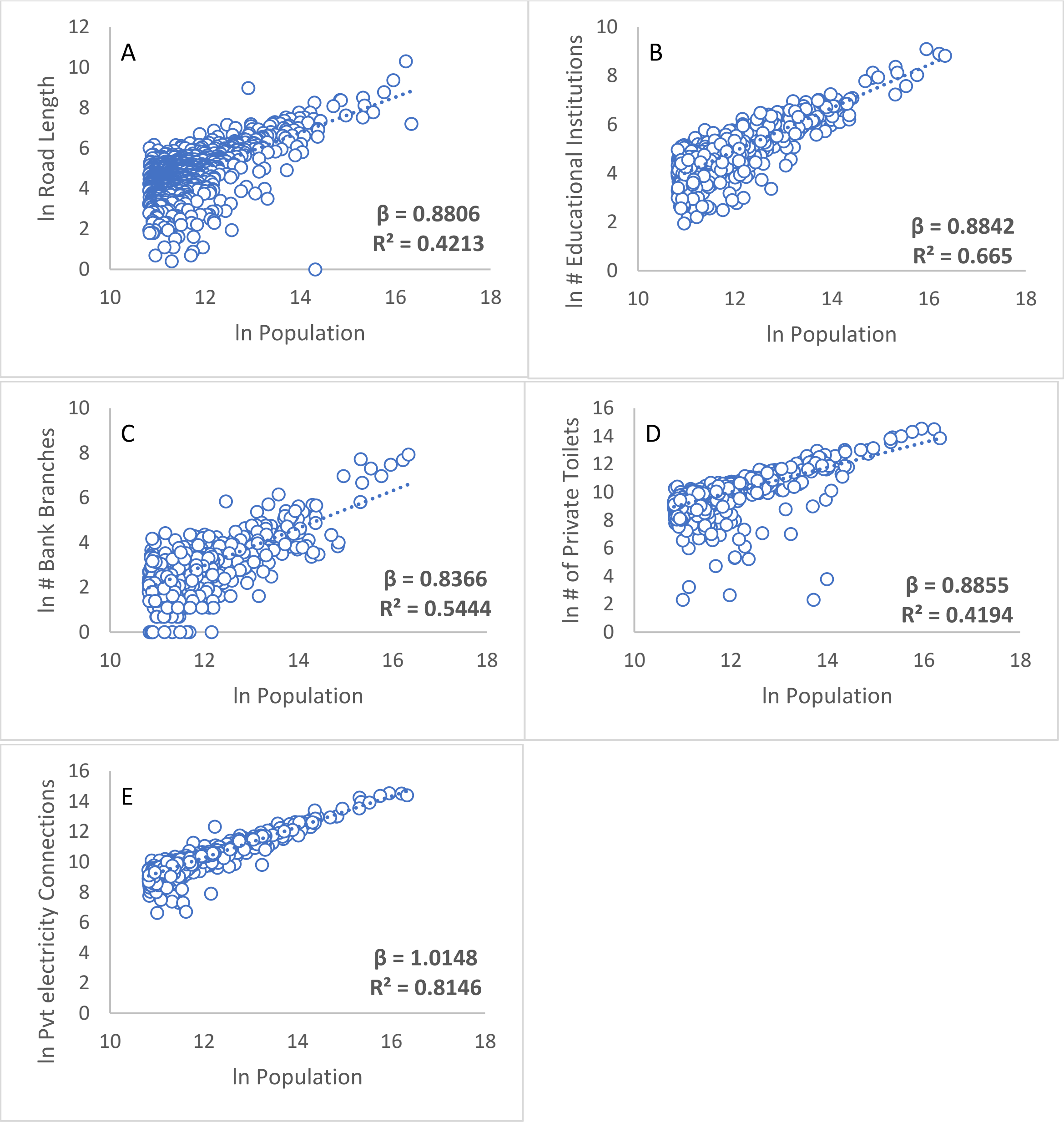

Figure B1: **Scaling of public and private infrastructure for individual cities with population.** Each panel shows the total value for each city (light blue circle) and the scaling best fit line. Urban Indicators shown are A. Road Length, B. Educational Institutions, C. Bank Branches, D. Private Toilets and E. Private Electricity Connections.

Appendix C: Challenges in urban GDP analysis

This lack of official urban GDP statistics has generated several estimates from non-official sources (details in Appendix A) – one from 2008 covering 13 cities measuring GDP in Purchasing Power Parity (PPP) terms and the other from 2010 covering 9 cities only, measuring nominal GDP, see Figure C1. These data sets are inconsistent with each other, one suggests a superlinear scaling with $\beta \simeq 1.12$ (roughly in line with other nations and theory), while the other suggests a slightly sublinear relationship with $\beta \simeq 0.95$. Other data sources provide partial proxies for testing the hypothesis of higher value economic activity in cities. For instance, the number of commercial and industrial electricity connections (not power usage) shows superlinear scaling with city size, with an exponent of $\beta \simeq 1.08$. In the absence of larger and more reliable datasets, it is difficult to say which of these relationships reflect the reality of GDP scaling, and the strength of economic agglomeration, in urban India.

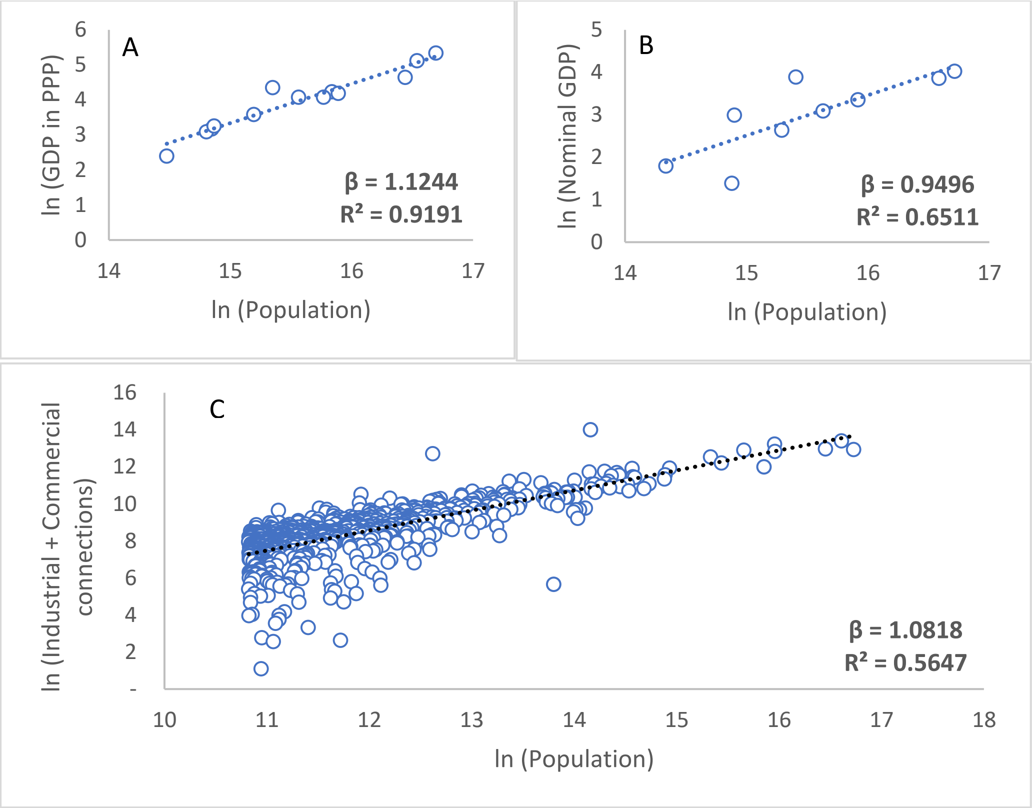

***Figure C1*: Scaling of proxies for economic activity with population from several partial sources and proxies**. A: GDP estimate with international Purchasing Power Parity for 13 cities estimated by PwC (in USD). B: Nominal GDP (in USD) estimated for 9 cities by McKinsey (8 of which are also contained in the PwC dataset, but with vastly different estimates in many cases). C: GDP proxied by the total numbers of industrial and commercial electricity connections in Indian cities from the Census of India 2011.

Appendix D: Sensitivity of scaling exponent for technological innovation to binning

In the main text, we use logarithmic binning with 9 bins to estimate the scaling exponent for technological innovation (base case). In this section, we use a number of techniques to establish the scaling relationship and assess the sensitivity of the exponent, Figure D1. We use logarithmic binning with a larger number of bins (19) and find that the scaling exponent, $\beta \simeq 1.49$ (with a 95% Confidence Interval (CI) of [1.18,1.80]), which is statistically close to the values obtained with 9 bins - $\beta \simeq 1.53$ with 95% CI of [1.22, 1.83]. One of the problems with logarithmic binning is the significant variations in bin sizes (with small bin sizes at the low and high ends of the scale and significantly larger bins in the middle), and to address this, we create 16 equisized bins of 20 data points each and compute the average population and patents for these bins. The scaling exponent using equisized bins is $\beta \simeq 1.55$ (with 95% CI of [1.26,1.84]). Finally, in an attempt to use each of the individual data points to assess the scaling exponent, we use a simplistic approach of adding 1 to the number of patents for each city (this ensures no zero values and systematically increases patents by 1 across the board) and find that under this method, the scaling exponent, $\beta \simeq 1.55$, with a 95% CI of [1.39,1.70]. Overall, under all these distinct estimates, the value of the scaling exponent and corresponding CIs does not show significant statistical variation from that obtained using the baseline logbinning approach with 9 bins.

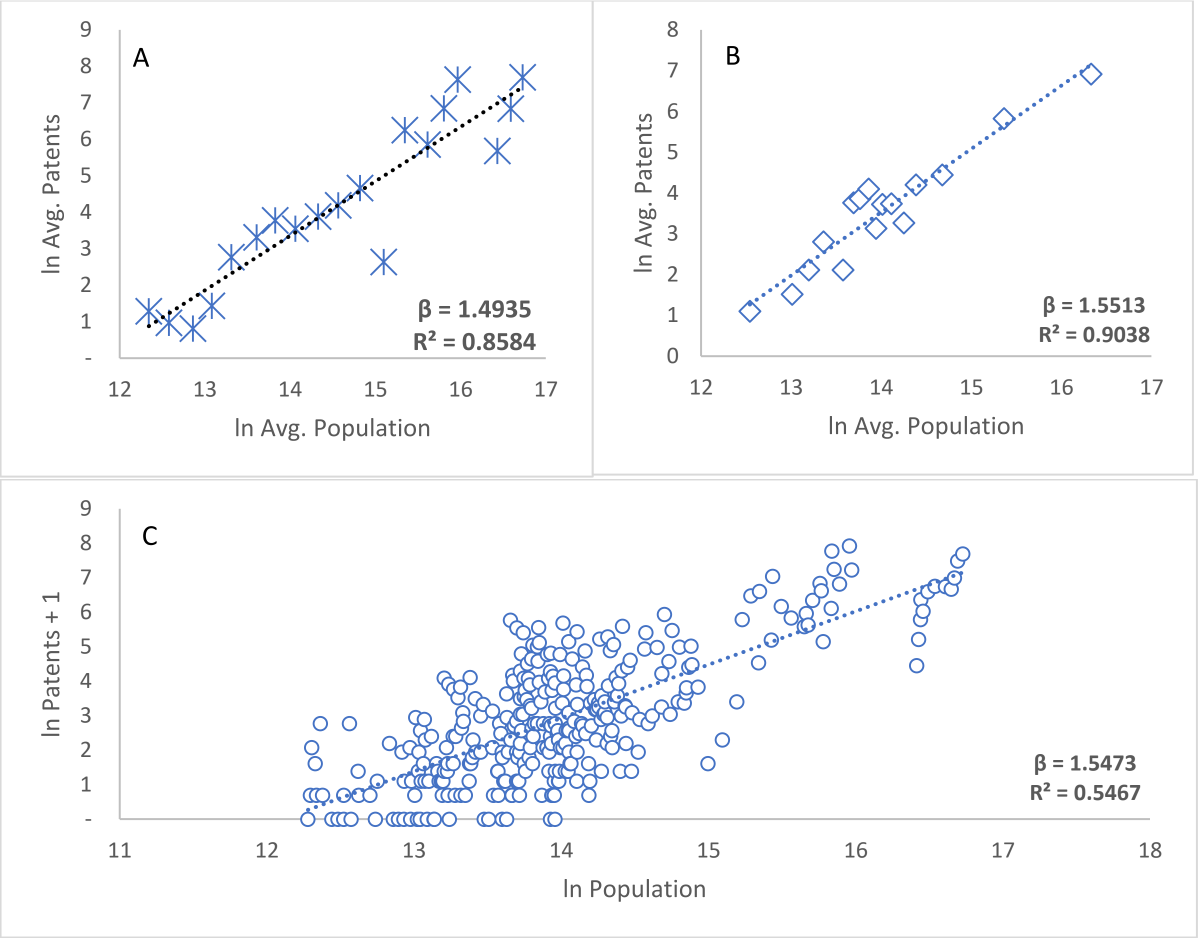

***Figure D1*: Scaling of Patents with population using different estimation techniques**. A: Logarithmic binning with 19 bins yields a scaling exponent of 1.4935 (95% CI: [1.18,1.80]). B: Binning data with 16 bins of equal size yields a scaling exponent of 1.5513 (95% CI: [1.26,1.84]). C: Adding 1 to patents across the board yields a scaling exponent of 1.5473 (95% CI: [1.39,1.70]). Under all cases, the value of the exponent does not show significant statistical variation from the base case (β = 1.5253 with 95% CI: [1.22,1.83]).
